# Impact of Comorbidities on Prognosis and Treatment Outcomes in Elderly Patients with Hodgkin Lymphoma

**DOI:** 10.3390/clinpract15010015

**Published:** 2025-01-13

**Authors:** Dávid Tóthfalusi, Boglárka Dobó, Fanni Borics, László Imre Pinczés, Árpád Illés, Zsófia Miltényi

**Affiliations:** 1 Division of Haematology, Department of Internal Medicine, Faculty of Medicine, University of Debrecen, 4032 Debrecen, Hungary; dobo.boglarka@med.unideb.hu (B.D.); borics.fanni@med.unideb.hu (F.B.); pinczes.laszlo.imre@med.unideb.hu (L.I.P.); illes.arpad@med.unideb.hu (Á.I.); miltenyi.zsofia@med.unideb.hu (Z.M.); 2 Doctoral School of Clinical Medicine, University of Debrecen, 4032 Debrecen, Hungary

**Keywords:** Hodgkin lymphoma, elderly patients, prognosis, survival chances

## Abstract

**Background/Objectives**: Hodgkin lymphoma (HL) primarily affects young adults, but about 20% of cases occur in patients over the age of 60 years. Older individuals often have comorbidities and poorer functional status, which can affect treatment choices. **Methods**: We retrospectively analyzed data from HL patients over 60 years old who were treated at our institution between January 2010 and December 2023. We examined various factors, such as blood parameters (e.g., platelet count, lactate dehydrogenase (LDH), C-reactive protein (CRP)), PET/CT results and comorbidities (e.g., hypertension, diabetes, cardiovascular diseases), to assess their impact on overall survival (OS) and progression-free survival (PFS). Diagnostic efficiency was determined via receiver operating characteristic analysis, while the survival outcomes were evaluated using the Cox proportional hazards model. **Results**: A total of 35 patients with a median age of 68 were treated. The most common subtype was nodular sclerosis, and 72% of patients were in advanced stages at diagnosis. Treatment varied by age, with younger patients receiving ABVD and older patients (80–89) receiving brentuximab vedotin with dacarbazine. The survival of older patients, when analyzed by age groups, did not show a significant difference in the OS (*p* = 0.16) and PFS (*p* = 0.11). Comorbidities significantly worsened survival, with patients who scored > 7 on the Charlson Comorbidity Index (CCI) showing a 5-year PFS of 41.3%, compared to 91.3% for those who scored ≤ 7. Among the tested laboratory parameters, a platelet count over 310.5 G/L and an absolute lymphocyte count below 0.47 G/L were found to be independent risk factors for OS. Patients with neither or only one of these risk factors demonstrated a 5-year OS of 81.7%, whereas those presenting with both risk factors experienced a reduced 5-year OS of 70%. For PFS, a white blood cell count > 8.48 G/L, a platelet count > 310.5 G/L, and advanced age (>73.5 years) were identified as significant adverse prognostic factors. Patients with none of these risk factors had a 5-year PFS of 100%, whereas those with ≥ 1 risk factor had a 5-year PFS of 35.6%. **Conclusions**: Comorbidities play a greater role in prognosis than chronological age, emphasizing the need for personalized treatment approaches.

## 1. Introduction

Hodgkin lymphoma (HL) is a malignancy involving the lymph nodes and the lymphatic system. Most patients are diagnosed in early adulthood (especially in their 20s), followed by another peak with patients over the age of 60 years, which represents almost 20% of all cases [1]. Although the treatment for HL is highly effective, an older age is associated with an inferior clinical course attributed to multiple factors. Older patients often have multimorbidity and poor functional status, which may affect the choice and toleration of treatment [2]. Histologic and biologic differences (mixed cellularity subtype, Epstein–Barr virus (EBV)-related disease), less ability to tolerate chemotherapy and treatment-related complications also lead to poorer survival chances [1,3].

Advancements in medical science have significantly increased life expectancy over recent decades. As a consequence, the population of older patients has increased significantly. An accurate geriatric assessment of comorbidities and individual functional status is needed to evaluate a person’s health and well-being. It provides information for the decision-making process, avoiding mistakes in treatment based on only the chronological age [4]. With the help of these scores, we have the opportunity to define patients’ prognosis, functional status and fitness for treatment. A fitness-based treatment choice would be useful to see who will benefit from full, curative-intention treatment and who will benefit from alternative solutions in order to enhance quality of life, treatment tolerance and recovery [5].

Impaired abilities are one of the key factors used to assess survival outcomes. These scores evaluate an individual’s disability by utilizing the concepts of the activities of daily living (ADL) and instrumental activities of daily living (IADL). The ADL comprise activities indispensable for basic daily activities (bathing, dressing, eating). ADL assessments help determine a patient’s physical independence and ability to tolerate treatments, particularly aggressive therapies. Patients need treatment plans that consider their functional limitations and potential need for supportive care. The total score varies between six (maximum performance) and zero (lack of performance). The IADL refer to the skills required for independent interaction within the community, such as using the telephone, shopping, managing household tasks, doing laundry, and handling finances. IADL assessments provide insight into a patient’s cognitive and physical ability to manage complex tasks and maintain independence. This information is crucial for planning post-treatment recovery and determining if additional support services are needed. The summary score ranges from zero (low function, dependent) to eight (high function, independent) [6].

The presence of comorbidities is associated with decreased life expectancy. Different indexes have been applied to assess the survival outcomes and therapeutic complications due to pathological conditions. The Charlson Comorbidity Index (CCI) score estimates the 10-year survival rate in patients with multiple comorbidities (cardiac, neurological, renal, malignancy, immunodeficiency). In HL patients, it is used to predict treatment tolerance, potential complications and overall survival outcomes [7]. The Cumulative Illness Rating Scale-Geriatric (CIRS-G) was created to help quantify the burden of chronic disease in geriatric patients and inform about patients’ potential for treatment and recovery [8].

The treatment of HL has developed over the last decade and the disease has become one of the most curable malignancies in young patients. Unfortunately, the outcomes in older patients are still poor. HL often presents as a more aggressive disease with B symptoms and an advanced stage at diagnosis. Treating patients over 60 years old comes with difficulties. Older patients have more comorbidities and poorer tolerability of therapy, which lead to increased toxicity and higher treatment-related mortality [9]. The treatment selection should be not only based on the age of the patient, as we should assess the functional status and comorbidities with the help of a geriatric assessment. Older but fit patients should be treated with curative intention similar to younger patients. The treatment for unfit patients must be highly individualized, and lower-intensity chemotherapy should be considered [1].

The aim of this research was to evaluate the treatment outcomes of HL patients over the age of 60 years at our clinic and to identify factors that could guide therapeutic decisions. To achieve this, we analyzed a broad set of data to investigate the relationship between patient- and disease-related characteristics and their influence on survival outcomes. We specifically sought to assess the utility of geriatric scales, including the CCI, as predictors of survival and to understand their role in optimizing treatment strategies.

This study fills a critical gap in the literature by being one of the first to examine the combined effects of prognostic markers and geriatric assessment tools in this population. HL is typically curable in younger patients; however, the outcomes for older patients remain poor due to the high prevalence of comorbidities and reduced treatment tolerance. Despite the availability of emerging therapies, such as brentuximab vedotin (BV) and programmed death receptor-1 (PD-1) inhibitors, existing research has not adequately explored how these treatments interact with age-related factors. Additionally, while clinical trial data on these therapies exist, they often fail to reflect everyday clinical practice and accessibility, which vary between countries. Notably, this study investigated the use of BV through real-world data, providing valuable insights into its practical application.

The insights from this study are intended to address this gap, offering evidence-based guidance to improve clinical decision-making. Specifically, we advocate for integrating geriatric assessments into routine practice to tailor treatment plans for elderly HL patients, thereby improving their survival outcomes and quality of life.

## 2. Materials and Methods

We conducted a retrospective analysis of data from HL patients over the age of 60 years, who were diagnosed and treated at our institution between 1 January 2010 and 31 December 2023. This study was performed in compliance with the Declaration of Helsinki and received approval from the Institutional Review Board (or Ethics Committee) of the University of Debrecen. Patients were selected based on a confirmed HL diagnosis and the availability of complete clinical data. To ensure a comprehensive analysis, we categorized the patients into three age groups: 60–69, 70–79, and 80–89 years. The rationale behind this categorization was that the number of comorbidities tends to increase with advancing age. This stratification allowed us to observe differences in tolerability and survival outcomes across varying levels of age-related vulnerability. We analyzed several parameters (laboratory parameters: blood count, lactate dehydrogenase (LDH), C-reactive protein (CRP), thymus and activation-regulated chemokine (TARC) level at diagnosis, after two cycles of treatment and at the end of the treatment; positron emission tomography–computed tomography (PET/CT) results: Deauville score; comorbidities: hypertension, diabetes mellitus, cardiovascular and cerebrovascular diseases, nephropathy, solid neoplasm) as prognostic factors with an impact on survival chances. The diagnostic efficiency of various independent variables was assessed using receiver operating characteristic (ROC) analysis, followed by a calculation with the Youden Index. The effect of the variables on the endpoints (overall survival—OS, progression-free survival—PFS) was assessed using the Cox proportional hazards regression model. A stepwise Cox proportional hazards model was built for multivariable survival analysis, with covariates that were significant in the univariable analysis entered in a hierarchical fashion using forward selection (*p* < 0.5 for inclusion). OS was defined as the time from the diagnosis of HL to death from any cause. PFS was defined as the time from the completion of first-line treatment for HL to the progression of the disease or death from any cause.

We routinely use the European Organisation for Research and Treatment of Cancer (EORTC, Brussels, Belgium) prognostic classification in early-stage disease; based on this, patients are divided into two categories: early-stage favorable (ESF) and early-stage unfavorable (ESU). In advanced stage (ADV) disease, the Hasenclever–Diehl score is used. In daily practice, the German Hodgkin Study Group (GHSG, Cologne, Germany) prognostic score is also determined if only one or two nodal sites are involved. Thus, we wanted to select the very-early-stage favorable group (none of the following are present: more than two involved sites, mediastinal mass ratio > 0.33, extranodal extension, and an erythrocyte sedimentation rate (ESR) > 50, or ESR > 30 if B-symptoms are present), as the therapy used in their case will be different. The EORTC factors are not suitable for this, as people over the age of 50 already belong to the unfavorable group [10]. The CCI was used to evaluate the burden of comorbidities in this cohort. This tool assigns weighted scores to various comorbid conditions, predicting the 10-year survival probabilities. A higher CCI score indicates worse prognostic outcomes. The CCI’s reproducibility and predictive value make it a valuable addition to geriatric assessments in elderly HL patients.

In everyday practice at our clinic, for the treatment of those fit patients who are under the age of 70 years, we give treatment with a curative intent, using doxorubicin, bleomycin, vinblastine and dacarbazine (ABVD) chemotherapy treatment that is similar to the treatment of younger patients. Patients with newly diagnosed classic HL undergo a baseline PET/CT scan, receive two cycles of ABVD chemotherapy, and then undergo an interim PET/CT scan. The exclusion of bleomycin from the ABVD regimen following a negative interim PET/CT scan after two cycles of ABVD seems to be safe, with no significant reduction in efficacy in advanced-stage disease. The treatment for patients with very-early-stage favorable disease (based on the GHSG scores) involves a combination of two cycles of ABVD, followed by involved site radiotherapy (ISRT) with 20 gray. For patients with ESU disease, the treatment consists of two cycles of ABVD, followed by two or four cycles of ABVD or AVD (omission of bleomycin)—based on the interim PET/CT result—or two cycles of ABVD and ISRT with 30 gray [11]. The therapy for unfit or frail patients is less clear and should be individualized with the use of lower-intensity treatment methods such as BV.

## 3. Results

A total number of 35 patients over sixty years of age were treated, with a median age of 68 (range: 60–88) years. There were 21 patients under the age of 70 years, 9 patients aged between 70 and 79 years, and 5 patients older than 80 years. Nodular sclerosis was the most common histological subtype, occurring in 43% of patients, and 66% of the patients had B symptoms. According to the EORTC and GHSG scores, at the time of diagnosis, 72% of the patients were in an advanced stage (Table 1). Moreover, 23% of the patients relapsed, and 26% died (with 33.3% dying from the underlying disease).

### 3.1. Treatment Methods Based on Age Groups and Comorbidities

Under the age of 70 years, 100% of patients received ABVD-like (using epirubicin instead of doxorubicin) treatment; among those aged 70–79 years, 56% of patients received ABVD-like treatment; and 60% over the age of 80 years received BV plus dacarbazine (DTIC) treatment (Table 2).

Nearly 90% of the patients were found to have at least one form of comorbidity. Cardiovascular diseases were the most common, occurring in 69% of cases, but diabetes and chronic renal failure were also frequently observed among the comorbidities. Additionally, 23% had another solid tumor (there was no typical tumor type).

Under the age of seventy years, we treated with a curative intent, and there was no contraindication for ABVD-like therapy. Four of these patients passed away. One of them died of COVID-19 infection, with an active underlying disease. Another patient was hospitalized due to pneumonia and urinary tract infection, and the cause of death was complicated sepsis. The other two patients died in non-hospital conditions, so we have no information about them.

In the 70–79 age group, we administered ABVD-like therapy to five patients, three of whom died. One of them died of cardiorespiratory failure due to severe heart failure. The second patient died from severe pneumonia, while the cause of death for the third patient is unknown. Two patients received epirubicin, vinblastine, dacarbazine (EVD) therapy; one of them had an ECOG 2 status, with a history of ischemic heart disease and stroke. Regrettably, pneumonia developed during treatment, and we lost this patient while his disease was still active. The other patient had known hypertension, diabetes mellitus and polymyalgia rheumatica; she tolerated the treatment well and has been in complete remission for six years. We chose the BV + DTIC treatment as the first line for two patients, one of whom stopped after six cycles due to side effects and is currently in remission; our other patient was unfortunately refractory to the treatment and has been receiving PD-1 inhibitor treatment since then.

Among the patients aged 80–89 years, we administered ABVD therapy in only one case; this patient was undergoing treatment in 2010 and at that time when we had no other therapeutic options. One patient received EVD therapy, which we could administer for only one full cycle due to the patient’s general condition, and then, after 18 months, her disease progressed. At that point, we used BV-DTIC, followed by PD-1 inhibitor therapy, and five years after diagnosis, the patient is still alive. Three patients received BV-DTIC therapy; one was heavily pre-treated (for breast cancer and marginal zone lymphoma), while another patient’s compliance was inadequate and we discontinued this therapy too. Our third patient was the oldest in the study (88 years old), so we did not recommend a stronger/more aggressive treatment.

### 3.2. Survival Outcomes According to Age Groups and Comorbidities

**Figure 1:**
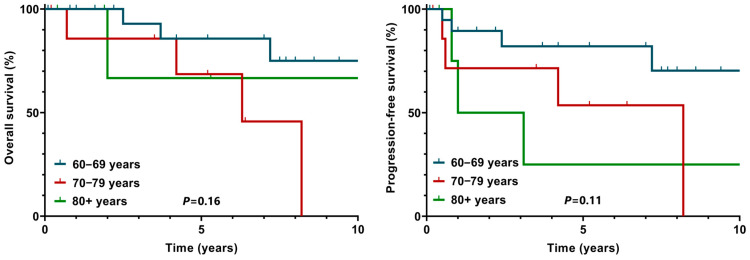
The survival outcomes of patients with Hodgkin lymphoma analyzed by the age groups.

The survival of older patients was not significantly worse when analyzed by the age groups (Figure 1).

**Figure 2:**
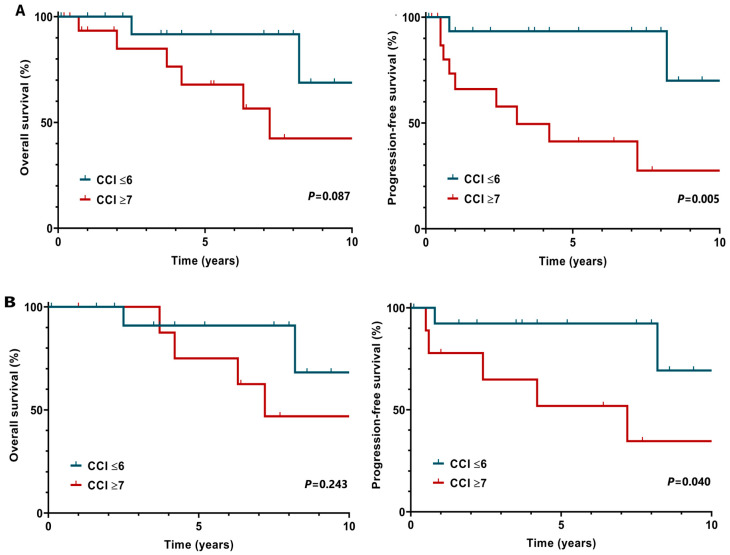
(**A**,**B**) Survival chances based on the Charlson Comorbidity Index (CCI) (**A**) and the CCI with the combination of ABVD treatment (**B**).

Comorbidities considerably reduced survival chances. According to the CCI, patients with more than 7 points had significantly worse 5-year PFS (91.3% vs. 41.3%, *p* = 0.005). We observed the same for those patients who had >7 points and received ABVD-like treatment (90.9% vs. 51.9%, *p* = 0.040) (Figure 2A,B).

### 3.3. Laboratory Parameters and Survival

To identify independent prognostic factors for OS and PFS, a stepwise Cox proportional hazards regression model was employed. Laboratory parameters with significant associations in the univariate analysis were subsequently included in a multivariable model using forward selection (*p* < 0.5 for inclusion). This approach allowed for the identification of independent predictors (Table 3).

**Figure 3:**
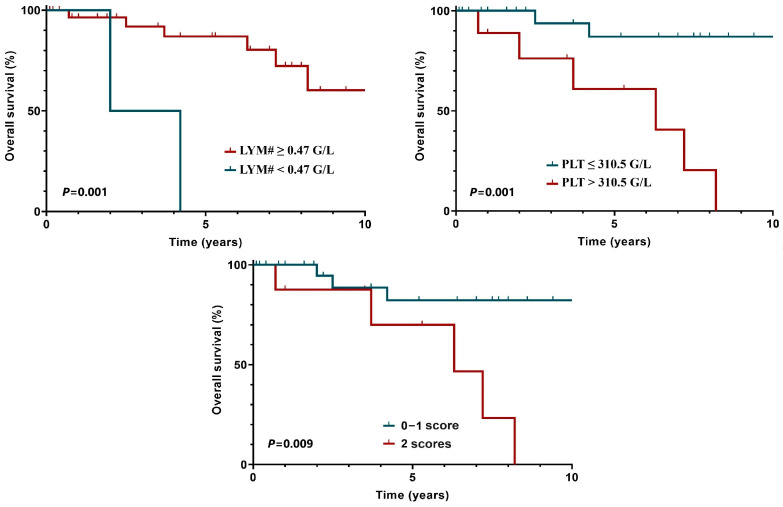
Risk factors for overall survival (PLT: platelet count; LYM#: absolute lymphocyte count).

Among the tested laboratory parameters, a platelet count over 310.5 G/L [150–400 G/L] and a low absolute lymphocyte count < 0.47 G/L [0.9–3.1 G/L] were identified as independent risk factors for OS. The optimal cut-off level was determined using the receiver operating characteristic method. Each parameter, both individually and in combination, significantly affected OS (Figure 3). Based on this, the 5-year OS of patients with a score of 0 or ≤1 was 81.7%, while the 5-year OS of patients with a score of 2 was 70%. In the case of the other investigated laboratory parameters, no significant differences were found in terms of OS.

**Figure 4:**
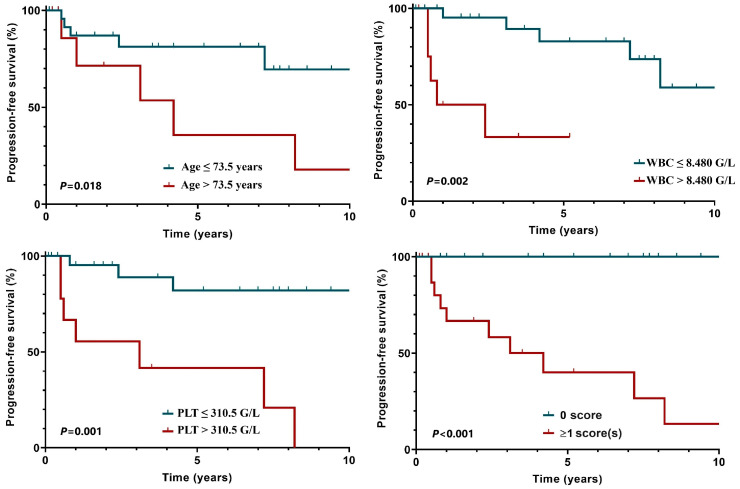
Risk factors for progression-free survival (WBC: white blood cell count; PLT: platelet count).

A white blood cell count (WBC) over 8.48 G/L [4.5–10.8 G/L], a platelet count over 310.5 G/L and an advanced age (>73.5 years) were identified as significant adverse prognostic factors for PFS. Each of these parameters, whether considered separately or in combination, had a significant impact on PFS (Figure 4). According to these results, those who did not fulfill any of the requirements had a 5-year PFS of 100%, while the 5-year PFS of patients with a score ≥1 was 35.6%.

## 4. Discussion

The poor prognosis of HL in elderly patients stems from their inability to tolerate conventional treatments because of their poor performance status or age-related comorbidities. The use of geriatric scores provides the opportunity to choose the most suitable treatment method based on the patient’s fitness status.

The Italian Lymphoma Foundation (FIL) utilizes a geriatric assessment that involves the following factors: (1) age (≥80 vs. <80 years), (2) comorbidities (based on the CIRS-G score), ADL and IADL. Patients are classified into three categories: fit, unfit and frail. Fit patients are individuals under 80 years who have minimal or no limitations in the ADL (score ≥ 5) and IADL (score ≥ 6), and without significant comorbidities. Unfit patients are those who are over 80 years old, independent (ADL = 6, IADL = 8) and have no comorbidities, or those under the age of 80 years with significant functional impairments (ADL < 5, IADL < 6) and at least one comorbidity (with a score of 3–4) or more than eight comorbidities with a score ≥ 2. Frail patients include individuals aged 80 or older who are dependent in terms of multiple ADLs with a score of less than 6, IADLs with a score of less than 8, and/or have significant comorbidities (at least one comorbidity with a score of 3–4, or five or more comorbidities with a score of 2 or higher). This method provides information for determining the appropriate treatment strategy and dosage intensity. It separates a subgroup of older adults under the age of 80 years who could potentially benefit from curative treatment using full-dose therapy, achieving similar outcomes to younger patients. Frail patients with multiple comorbidities and impairments in the ADL/IADL may not tolerate aggressive treatment and may experience comparable outcomes with a palliative approach [5,12].

Despite the fact that this geriatric assessment can help to choose the proper treatment for elderly patients, it is not completely reliable. Patients over the age of eighty years are automatically classified in the unfit category; on the other hand, even with a loss of one point, they can easily be classified in the unfavorable category. Under the age of 70 years, we use curative therapy; over 70 years, we make a personalized decision based on the general condition and comorbidities.

### 4.1. Treatment Options for HL at Different Stages

The standard treatment approach for individuals with early-stage favorable disease involves a combination of two cycles of ABVD, followed by ISRT with 20 gray [11]. Another treatment method is using 2–4 cycles of cyclophosphamide, doxorubicin, vincristine, prednisone (CHOP), followed by radiotherapy, which is a well-tolerated and effective treatment for elderly patients with HL [12].

For patients with early-stage unfavorable disease, the recommended treatment consists of two cycles of ABVD, followed by four cycles of AVD and ISRT with 30 gray [13]. ABVD treatment is notably more toxic for older patients, with a higher incidence of bleomycin-related lung toxicity in this population. A French study involving 147 HL patients over 60 years old found that bleomycin-related toxicity led to dose reduction in more than a third of patients [14]. The GHSG study group, in their HD10 and HD13 trials, randomized older ESF HL patients to receive either two or four cycles of ABVD or two cycles of AVD, followed by ISRT. Four cycles of ABVD were associated with significant additional toxicity and mortality. It is generally recommended that if ABVD is used in older patients, no more than two cycles should include bleomycin [9,15]. We also apply this principle at our clinic, but we rarely supplement the treatment with personalized radiation therapy. If the patient receives chemotherapy, bleomycin is omitted in case of a negative interim PET-CT result.

The therapy for fit older patients with advanced disease can consist of two cycles of ABVD, followed by four cycles of AVD if the FDG-PET scan is negative after two cycles of ABVD [16]. We use the same treatment method for advanced-stage disease. Six cycles of CHOP, with or without ISRT, might be another possible treatment option for patients with advanced disease [12].

According to a study, the combination of BV and DTIC is a well-tolerated and highly effective treatment method in elderly, frail patients. Despite these patients being older and having more advanced disease, the results showed better tolerability and effectiveness compared other chemotherapy options in elderly patients. Patients received 12 cycles of this combination. For patients treated with BV plus DTIC, the objective response rate (ORR) was 100% and 62% of patients achieved complete remission (CR). The median PFS was 17.9 months [17]. We also use this combination in frail patients, measuring the response with PET/CT after six cycles.

Based on results of the ECHELON-1 trial, BV in combination with AVD is increasingly used for the treatment of older, less fit patients with advanced-stage HL. Effective and tolerable treatments are essential for older patients with HL. Peripheral neuropathy (PN) was the most common and disabling side effect of BV observed in clinical trials. In a phase II multicenter study, sequential treatment involving two cycles of BV, followed by six cycles of AVD, showed promising results. Although it is an available option, we have not yet tried it in elderly patients [18,19].

Nivolumab, an immune checkpoint inhibitor, has demonstrated significant efficacy in elderly HL patients. A randomized, phase 3 trial, S1826, was conducted by the National Clinical Trials Network to evaluate nivolumab-AVD (N-AVD) and BV-AVD in patients with advanced-stage HL patients over the age of 60 years. At a median follow-up of 2.1 years, the study found that N-AVD was superior to BV-AVD. Specifically, the 2-year PFS rates were 89% for N-AVD and 64% for BV-AVD. The hazard ratio (HR) was 0.24 (95% CI, 0.09 to 0.63), with a stratified one-sided log-rank *p*-value of 0.001. The 2-year OS rate was 96% for N-AVD, compared to 85% for BV-AVD. The HR was 0.16 (95% CI, 0.03–0.75), with a one-sided log-rank *p*-value of 0.005. The 2-year EFS rates were 89% for N-AVD and 58% for BV-AVD. The HR was 0.18 (95% CI, 0.07–0.47), with a one-sided log-rank *p*-value of 0.001. Early treatment discontinuation occurred in 10% of N-AVD patients and 33% of BV-AVD patients, with the primary reasons being adverse events (two vs. seven patients) and death (one vs. five patients). These results support the potential of N-AVD as a more effective and better-tolerated regimen for elderly patients with advanced-stage HL, highlighting significant improvements in PFS and EFS, and a trend toward improved OS compared to BV-AVD [20,21]. Although it is a promising treatment method, it is not yet available in everyday practice.

BV plus nivolumab is an emerging treatment option for older HL patients with comorbidities. This combination has been evaluated in various studies and clinical trials, particularly focusing on older or frailer patients who cannot tolerate standard treatment. It represents a promising treatment approach with better tolerability. Further long-term studies are needed to confirm its efficacy and safety in this unique population, but the early results are encouraging [22].

Unfortunately, HL patients with a low ejection fraction (EF) are frequently excluded from clinical trials, limiting our understanding of how to manage these individuals. A first real-world evidence study of HL patients with a low EF found that most of these patients were treated with an anthracycline (AC)-based chemotherapy. According to this study, PFS improved for patients who received AC-based therapies [23]. Anthracycline-based treatment would be important if the patient could tolerate it.

ABVD is a standard treatment for HL across age groups. The use of ABVD in 100% of our patients under the age of 70 years, and decreasing usage with increasing age, reflects common practice. Age significantly influenced the treatment decisions and outcomes. Patients under 70 years tolerated ABVD-like regimens better, often achieving favorable survival outcomes. In contrast, patients over 80 years frequently required less-intensive therapies, such as BV combined with DTIC, due to comorbidities and the potential for treatment-related toxicity. Treatment tolerability is highly variable, underscoring the need for predictive biomarkers to guide therapeutic choices. While age is an important consideration, it is not the sole determinant of treatment selection at our clinic. The decision is primarily influenced by comorbidities and their associated risks, such as cardiotoxicity from doxorubicin or pulmonary toxicity from bleomycin. For some patients, ABVD is not initiated due to these risks, while in others, the regimen is modified. Reducing ABVD to AVD is often deemed clinically acceptable, especially for older patients, where guidelines suggest limiting bleomycin to no more than two cycles due to its pulmonary toxicity. In advanced-stage disease, treatment typically continues with AVD beyond the initial cycles of ABVD. This variability in therapeutic approaches highlights the importance of individualized treatment planning based on patient-specific factors, including comorbidities and tolerance levels, to optimize outcomes in elderly patients.

The integration of novel therapies such as BV and PD-1 inhibitors into treatment algorithms for elderly HL patients represents a significant advancement. BV has demonstrated particular efficacy and tolerability in frontline settings, especially when combined with DTIC for frail patients who cannot undergo intensive chemotherapy regimens [17]. Clinical trials like ECHELON-1 have shown that BV combined with AVD can reduce treatment-related toxicity while maintaining efficacy. Although ECHELON-1 primarily focused on broader HL populations, subgroup analyses suggest potential benefits for older patients, particularly in balancing efficacy and tolerability [18,19]. PD-1 inhibitors like nivolumab are also transformative options for elderly patients, particularly those with significant comorbidities, due to their ability to modulate the immune response with a lower likelihood of severe adverse effects. Recent studies, including those evaluating nivolumab combined with AVD, have shown promising results in older patients, with emerging evidence suggesting improved outcomes in frontline settings for this demographic [21].

In practice, the treatment selection should be guided by careful consideration of comorbidities and the toxicity profiles of available therapies. In the relapsed or refractory setting, BV and PD-1 inhibitors offer critical options, with their integration based on prior treatments, patient fitness, and disease status. The incorporation of BV and PD-1 inhibitors into treatment algorithms for elderly HL patients represents a significant advancement in clinical oncology, providing more effective and tolerable treatment options. As clinical evidence continues to evolve, these therapies may become standard practice for elderly patients, offering hope for improved survival outcomes and quality of life.

### 4.2. Prognostic Factors for Survival Outcomes in HL

In our study, we conducted an analysis of patients over 60 years with HL that provides valuable insights into the prognostic factors impacting this age group. Since we conducted a real-world analysis, we had no influence over the number of cases. Based on the post hoc power analysis of the statistical tests performed, all the results presented have adequate statistical power. Below is a detailed comparison of our findings with other studies focusing on similar prognostic factors, including laboratory parameters and comorbidities.

Comorbidities were prevalent (89%) in our study, predominantly cardiovascular conditions, diabetes and chronic renal failure. The CCI showed a significant impact on 5-year PFS, indicating worse outcomes with higher comorbidity burdens. Similar findings are reported in other studies, highlighting the adverse impact of comorbidities on survival in older HL patients. Studies by SWOG and EORTC emphasize the importance of assessing and managing comorbidities in treatment planning [20,24]. The findings highlight the essential role of comprehensive geriatric assessments in managing elderly HL patients. Integrating tools such as the CCI into routine care allows clinicians to evaluate treatment fitness and make informed decisions that balance curative intent with tolerability. The use of doxorubicin, a critical component of curative regimens, should be considered whenever possible, but only after thorough evaluation through geriatric scoring systems, including frailty assessments and the CCI. For frail patients who may not tolerate standard therapies, novel approaches such as BV and PD-1 inhibitors provide effective and less toxic alternatives. These advancements represent a paradigm shift, offering new hope for improving outcomes while minimizing treatment-related toxicity in this vulnerable population.

Fewer studies have been conducted to investigate the prognostic value of different laboratory parameters. Alcoceba et al. reported high LDH and low serum albumin levels in patients with advanced-stage HL between the ages of 27 and 43 years. By combing pre-treatment albumin and LDH, the lactate dehydrogenase-to-albumin ratio (LAR) may be a good prognostic factor for HL patients. In our study, the LAR was not a significant prognostic factor for OS or PFS in patients with HL over 60 years. In fact, we did not limit the study to patients with an advanced stage, which may be the reason we did not obtain the same results [25].

Numerous studies have explored the prognostic significance of the lymphocyte, neutrophil, and monocyte counts in HL. For instance, Lee et al. examined the prognostic value of the lymphocyte-to-monocyte ratio (LMR) at diagnosis in HL and its correlation with survival outcomes in patients with a median age of 67 years. Their findings indicated that a low LMR at diagnosis was associated with poorer PFS and OS [26]. Another study from the north-eastern region of Hungary identified a reduced absolute lymphocyte/monocyte ratio (LMR) in the peripheral blood as a poor prognostic indicator in HL. Combining the LMR with the interim PET/CT scan results offered greater prognostic value than the interim PET/CT alone. Both OS and PFS were significantly worse in patients with a lower LMR and positive interim PET/CT findings [27].

Similarly, Reddy et al. found that the pre-treatment neutrophil-to-lymphocyte ratio (NLR) and platelet-to-lymphocyte ratio (PLR) are significant prognostic indicators for disease progression in early-stage HL patients between 24 and 42 years. Both the NLR and PLR were significantly linked to worse freedom from progression in the univariate analysis [28]. In our study, we analyzed the prognostic value in relation to PFS and OS of these parameters, but there were no significant results.

The studies mentioned above examined the prognostic value of laboratory parameters in HL patients, but most of them examined adult patients under the age of 60 years. Our study is one of the studies that most extensively investigated the prognostic significance of different laboratory values in patients with HL over the age of 60 years.

The findings that a platelet count over 310.5 G/L and an absolute lymphocyte count below 0.47 G/L are independent risk factors for OS likely reflect their roles in the immune response and tumor microenvironment. Elevated platelet counts could indicate a pro-inflammatory or hypercoagulable state, while low lymphocyte counts may signify impaired immune surveillance. Together, these parameters provide insights into the systemic effects of HL and its progression. Future studies should investigate the molecular and cellular mechanisms linking these laboratory abnormalities to survival outcomes, potentially uncovering novel therapeutic targets.

For PFS, a white blood cell count over 8.48 G/L, a platelet count over 310.5 G/L and an advanced age (>73.5 years) were confirmed as significant adverse prognostic factors, which underscores the importance of assessing both the disease burden and host factors. Exploring interventions aimed at modulating the immune response in elderly patients with HL may help to improve survival outcomes in this population.

This age-related result differs from the age limit of 80 years found in the FIL classification system. According to the FIL system, HL patients over 80 years are categorized as unfit, regardless of comorbidities. The classification distinguishes a group of older adults under 80 years who may benefit from a curative approach using full-dose therapy, while identifying a frail subgroup over 80 years who likely lack the functional reserve to tolerate aggressive treatments. This age difference can be attributed to the lower life expectancy at birth in Hungary compared to Italy.

Future research should aim to validate these findings in larger, multi-center cohorts to enhance their generalizability. Investigating the integration of novel therapies, such as immune checkpoint inhibitors and targeted treatments, with these prognostic markers could further refine personalized treatment strategies. Additionally, longitudinal studies assessing the impact of geriatric assessment tools in conjunction with laboratory parameters could provide a holistic approach to optimizing care for elderly HL patients. It is also crucial to consider local factors, including the availability of and funding for new therapies, which can vary significantly across regions. These factors can influence treatment choices and outcomes, underscoring the importance of adapting treatment protocols to the specific healthcare settings and resources available in different countries.

## 5. Conclusions

While this study provides valuable insights, its retrospective design and small sample size (*n* = 35) limit the generalizability of the findings. Additionally, the long study duration (2010–2023) introduces potential biases due to evolving treatment protocols and diagnostic methods over time. In Hungary, novel therapies such as BV and PD-1 inhibitors only became available from 2020 under a special funding scheme, with prior access limited to clinical trials. As a result, it is challenging to determine the true impact of these therapies on treatment outcomes. Future studies should address these limitations by utilizing larger, multi-institutional cohorts and prospective designs. It is also important to highlight that access to and funding for novel therapies vary significantly across countries, potentially impacting treatment outcomes. Standardized treatment protocols and contemporaneous data collection would improve the reliability and broader applicability of future findings. In summary, these studies contribute valuable insights into the complex interplay of the clinical, pathological, and laboratory factors that influence the outcomes in older HL patients. While specific findings vary across studies, they collectively highlight the importance of personalized treatment approaches and careful consideration of prognostic markers in managing older HL patients. Currently, there is no consensus on how to integrate biological markers with established clinical prognostic factors into comprehensive prognostic scores. Identifying the most accurate parameters for predicting the prognosis for individual patients remains a significant challenge, as does recognizing the considerable subset of patients for whom standard treatment regimens are inadequate.

## Figures and Tables

**Table 1:** Clinical presentation of Hodgkin lymphoma over the age of 60 years.

**Age groups**	**N**	**%**
60–69	21	60
70–79	9	26
80–89	5	14
**Gender**	**N**	**%**
Male	16	46
Female	19	54
**Histopathologic subtype**	**N**	**%**
Nodular sclerosis	15	43
Mixed cellularity	13	37
Lymphocyte rich	5	14
Lymphocyte depleted	2	6
**Stage**	**N**	**%**
I–II	10	28
III–IV	25	72
**EORTC**	**N**	**%**
Early-stage favorable	0	0
Early-stage unfavorable	10	28
Advanced	25	72
**GHSG**	**N**	**%**
Early	5	14
Intermediate	5	14
Advanced	25	72
**B symptoms**	**N**	**%**
	23	66
**Extranodal involvement**	**N**	**%**
	19	54
**Splenic involvement**	**N**	**%**
	13	37
**Bulky mediastinal**	**N**	**%**
	0	0

EORTC: European Organisation for Research and Treatment of Cancer; GHSG: German Hodgkin Study Group.

**Table 2:** Comparison of treatment methods based on age groups.

Comparison of Treatment Methods Based on Age Groups.						
	60–69 Years		70–79 Years		80–89 Years	
	N	%	N	%	N	%
ABVD or EBVD	21	100	5	56	1	20
AVD or EVD	0	0	2	22	1	20
BV + DTIC	0	0	2	22	3	60

ABVD: doxorubicin, bleomycin, vinblastine, dacarbazine; AVD: omission of bleomycin; EBVD: epirubicin, bleomycin, vinblastine, dacarbazine; EVD: omission of bleomycin; BV: brentuximab vedotin; DTIC: dacarbazine.

**Table 3:** Prognostic value of laboratory parameters in relation to overall survival and progression-free survival.

Variable	OS Cox Univariate	OS Cox Multivariate	PFS Cox Univariate	PFS Cox Multivariate
Age group	0.178		0.027	0.048
Gender	0.980		0.534	
Histopathologic subtype	0.340		0.233	
Stage	0.297		0.084	
EORTC	0.297		0.084	
GHSG	0.297		0.084	
B symptoms	0.381		0.279	
Extranodal involvement	0.440		0.084	
Splenic involvement	0.816		0.595	
Bulky mediastinal	-		-	
Charlson Comorbidity Index	0.114		0.015	0.069
TARC1	0.311		0.068	
TARC2	0.069		0.021	
dTARC	0.329		0.110	
White blood cell count	0.028		0.007	0.014
Absolute neutrophil count	0.092		0.161	
Neutrophil %	0.016	0.188	0.234	
Absolute lymphocyte count	0.011	0.009	0.662	
Lymphocyte %	0.223		0.714	
Absolute monocyte count	0.297		0.146	
Monocyte %	0.372		0.269	
Hemoglobin	0.033	0.652	0.079	
Platelet count	0.006	0.004	0.004	0.030
Lactate dehydrogenase	0.165		0.048	0.276
C-reactive protein	0.013	0.349	0.288	

OS: overall survival; PFS: progression-free survival; EORTC: European Organisation for Research and Treatment of Cancer; GHSG: German Hodgkin Study Group; TARC: thymus and activation-regulated chemokine; TARC1: TARC level measured at initial staging; TARC2: TARC level measured at interim assessment; dTARC: TARC level measured at restaging.

## Data Availability

The data sets generated during the current study are available from the corresponding author on reasonable request.

## References

[1] Carter J., David K.A., Kritharis A., Evens A.M. (2020). Current Treatment Options for Older Patients with Hodgkin Lymphoma. Curr. Treat. Options Oncol..

[2] Jagadeesh D., Diefenbach C., Evens A.M. (2013). XII. Hodgkin lymphoma in older patients: Challenges and opportunities to improve outcomes. Hematol. Oncol..

[3] Björkholm M., Weibull C.E., Eloranta S., Smedby K.E., Glimelius I., Dickman P.W. (2018). Greater attention should be paid to developing therapies for elderly patients with Hodgkin lymphoma—A population-based study from Sweden. Eur. J. Haematol..

[4] Ghimire K., Dahal R. (2024). Geriatric Care Special Needs Assessment. StatPearls.

[5] Soverini G., Tucci A. (2022). Clinical geriatric assessment in older patients with lymphoma: A narrative review. Ann. Lymphoma.

[6] Beltz S., Gloystein S., Litschko T., Laag S., van den Berg N. (2022). Multivariate analysis of independent determinants of ADL/IADL and quality of life in the elderly. BMC Geriatr..

[7] Jerry Teng C.-L., Tan T.-D., Pan Y.-Y., Lin Y.-W., Lien P.-W., Chou H.-C., Chen P.-H., Lin F.-J. (2022). Prognostic Factors for Clinical Outcomes in Patients with Newly Diagnosed Advanced-stage Hodgkin Lymphoma: A Nationwide Retrospective Study. Cancer Control.

[8] Salvi F., Miller M.D., Grilli A., Giorgi R., Towers A.L., Morichi V., Spazzafumo L., Mancinelli L., Espinosa E., Rappelli A. (2008). A Manual of Guidelines to Score the Modified Cumulative Illness Rating Scale and Its Validation in Acute Hospitalized Elderly Patients. J. Am. Geriatr. Soc..

[9] Snauwaert S., Hende V.V., Janssens A., André M. (2021). How to treat classical Hodgkin’s lymphoma in older patients: Belgian expert opinion. Belg. J. Hematol..

[10] Akhtari M., Milgrom S.A., Pinnix C.C., Reddy J.P., Dong W., Smith G.L., Mawlawi O., Abou Yehia Z., Gunther J., Osborne E.M. (2018). Reclassifying patients with early-stage Hodgkin lymphoma based on functional radiographic markers at presentation. Blood.

[11] Fuchs M., Goergen H., Kobe C., Kuhnert G., Lohri A., Greil R., Sasse S., Topp M.S., Schäfer E., Hertenstein B. (2019). Positron Emission Tomography–Guided Treatment in Early-Stage Favorable Hodgkin Lymphoma: Final Results of the International, Randomized Phase III HD16 Trial by the German Hodgkin Study Group. J. Clin. Oncol..

[12] Kolstad A., Nome O., Delabie J., Lauritzsen G.F., Fossa A., Holte H. (2007). Standard CHOP-21 as first line therapy for elderly patients with Hodgkin’s lymphoma. Leuk. Lymphoma.

[13] Gunther J.R., Fanale M.A., Reddy J.P., Akhtari M., Smith G.L., Pinnix C.C., Milgrom S.A., Yehia Z.A., Allen P.K., Osborne E.M. (2016). Treatment of Early-Stage Unfavorable Hodgkin Lymphoma: Efficacy and Toxicity of 4 Versus 6 Cycles of ABVD Chemotherapy With Radiation. Int. J. Radiat. Oncol. Biol. Phys..

[14] Stamatoullas A., Brice P., Bouabdallah R., Mareschal S., Camus V., Rahal I., Franchi P., Lanic H., Tilly H. (2015). Outcome of patients older than 60 years with classical Hodgkin lymphoma treated with front line ABVD chemotherapy: Frequent pulmonary events suggest limiting the use of bleomycin in the elderly. Br. J. Haematol..

[15] Böll B., Goergen H., Behringer K., Bröckelmann P.J., Hitz F., Kerkhoff A., Greil R., von Tresckow B., Eichenauer D.A., Bürkle C. (2016). Bleomycin in older early-stage favorable Hodgkin lymphoma patients: Analysis of the German Hodgkin Study Group (GHSG) HD10 and HD13 trials. Blood.

[16] Barrington S.F., Kluge R. (2017). FDG PET for therapy monitoring in Hodgkin and non-Hodgkin lymphomas. Eur. J. Nucl. Med. Mol. Imaging.

[17] Friedberg J.W., Forero-Torres A., Bordoni R.E., Cline V.J.M., Patel Donnelly D., Flynn P.J., Olsen G., Chen R., Fong A., Wang Y. (2017). Frontline brentuximab vedotin in combination with dacarbazine or bendamustine in patients aged ≥60 years with HL. Blood.

[18] Evens A.M., Connors J.M., Younes A., Ansell S.M., Kim W.S., Radford J., Feldman T., Tuscano J., Savage K.J., Oki Y. (2021). Older patients (aged ≥ 60 years) with previously untreated advanced-stage classical Hodgkin lymphoma: A detailed analysis from the phase III ECHELON-1 study. Haematologica.

[19] Bowers J.T., Anna J., Bair S.M., Annunzio K., Epperla N., Pullukkara J.J., Gaballa S., Spinner M.A., Li S., Messmer M.R. (2023). Brentuximab vedotin plus AVD for Hodgkin lymphoma: Incidence and management of peripheral neuropathy in a multisite cohort. Blood Adv..

[20] Rutherford S.C., Li H., Herrera A.F., Leblanc M., Ahmed S., Davison K.L., Casulo C., Bartlett N.L., Tuscano J.M., Hess B. (2023). Nivolumab-AVD Is Better Tolerated and Improves Progression-Free Survival Compared to Bv-AVD in Older Patients (Aged ≥60 Years) with Advanced Stage Hodgkin Lymphoma Enrolled on SWOG S1826. Blood.

[21] Herrera A.F., LeBlanc M., Castellino S.M., Li H., Rutherford S.C., Evens A.M., Davison K., Punnett A., Parsons S.K., Ahmed S. (2024). Nivolumab+AVD in Advanced-Stage Classic Hodgkin’s Lymphoma. New Engl. J. Med..

[22] Cheson B.D., Bartlett N.L., LaPlant B., Lee H.J., Advani R.J., Christian B., Diefenbach C.S., Feldman T.A., Ansell S.M. (2020). Brentuximab vedotin plus nivolumab as first-line therapy in older or chemotherapy-ineligible patients with Hodgkin lymphoma (ACCRU): A multicentre, single-arm, phase 2 trial. Lancet Haematol..

[23] Annunzio K., McLaughlin E., Voorhees T., Shea L.K., Sumransub N., Rose A., Olszewski A.J., Bailey N., Patel K., Moyo T.K. (2023). Treatment Patterns and Outcomes for Patients with Classic Hodgkin Lymphoma (cHL) and Cardiomyopathy with Low Ejection Fraction (EF): Real-World Evidence (RWE) from 16 US Academic Centers. Blood.

[24] Vassilakopoulos T.P., Liaskas A., Pereyra P., Panayiotidis P., Angelopoulou M.K., Gallamini A. (2023). Incorporating Monoclonal Antibodies into the First-Line Treatment of Classical Hodgkin Lymphoma. Int. J. Mol. Sci..

[25] Reyes-Pérez E.N., Flores-Cuevas L.M., Martínez-Mier G., Chávez-Güitrón L.E., Martínez-Jiménez M.C., Audelo-Guzmán M., Calderón-García J., Reyes-Ruiz J.M. (2023). Predictive value of the lactate dehydrogenase-to-albumin ratio (LAR) in classical Hodgkin’s lymphoma. Ann. Blood.

[26] Kamiya N., Ishikawa Y., Kotani K., Hatakeyama S., Matsumura M. (2022). Monocyte-to-Lymphocyte Ratio in the Diagnosis of Lymphoma in Adult Patients. Int. J. Gen. Med..

[27] Simon Z., Barna S., Miltenyi Z., Husi K., Magyari F., Jona A., Garai I., Nagy Z., Ujj G., Szerafin L. (2016). Combined prognostic value of absolute lymphocyte/monocyte ratio in peripheral blood and interim PET/CT results in Hodgkin lymphoma. Int. J. Hematol..

[28] Reddy J.P., Hernandez M., Gunther J.R., Dabaja B.S., Martin G.V., Jiang W., Akhtari M., Allen P.K., Atkinson B.J., Smith G.L. (2018). Pre-treatment neutrophil/lymphocyte ratio and platelet/lymphocyte ratio are prognostic of progression in early stage classical Hodgkin lymphoma. Br. J. Haematol..

